# Analysis of the Molecular Signaling Signatures of Muscle Protein Wasting Between the Intercostal Muscles and the Gastrocnemius Muscles in *db/db* Mice

**DOI:** 10.3390/ijms20236062

**Published:** 2019-12-01

**Authors:** Kun Woo Kim, Mi-Ock Baek, Ji-Young Choi, Kuk Hui Son, Mee-Sup Yoon

**Affiliations:** 1 Department of Thoracic and Cardiovascular Surgery, Gachon University Gil Medical Center, College of Medicine, Gachon University, Incheon 21565, Korea; isee03@gilhospital.com; 2 Department of Molecular Medicine, School of Medicine, Gachon University, Incheon 21999, Korea; mioki9436@hanmail.net; 3 Lee Gil Ya Cancer and Diabetes Institute, Gachon University, Incheon 21936, Korea; jyjy31@hanmail.net; 4 Department of Health Sciences and Technology, GAIHST, Gachon University, Incheon 21999, Korea

**Keywords:** muscle atrophy, intercostal muscles, gastrocnemius muscle, mTOR, Akt, FoxO, Atrogin-1, MuRF1, autophagic flux

## Abstract

Type 2 diabetes (T2D) patients suffer from dyspnea, which contributes to disease-related morbidity. Although T2D has been reported to induce a catabolic state in skeletal muscle, whether T2D induces muscle wasting in respiratory muscles has not yet been investigated. In this study, we examine the difference in the molecular signaling signature of muscle wasting between the intercostal and gastrocnemius muscles using *db/db* mice, a well-known diabetic mouse model. Akt phosphorylation was significantly decreased in both the intercostal and gastrocnemius muscles of *db/db* mice and was accompanied by a decrease in mTORC1 activity. In addition, FoxO phosphorylation was suppressed, and ubiquitin-proteasome degradation, characterized by the level of Atrogin-1 and MuRF1, was subsequently enhanced in both muscle types of *db/db* mice. An increase in LC3BII levels and a decrease in p62 levels marked the occurrence of substantial autophagy in the gastrocnemius muscle but not in the intercostal muscles of *db/db* mice. Therefore, we suggest that the signaling events of muscle wasting in the intercostal muscles of *db/db* mice are different from those in the gastrocnemius muscle of *db/db* mice.

## 1. Introduction

Type 2 diabetes (T2D) is a chronic metabolic disease in which the body cannot use insulin effectively [1]. T2D is characterized by hyperglycemia, hyperinsulinemia, high plasma levels of free fatty acids (FFAs), and insulin resistance. Dyspnea, a shortness of breath, has been observed in patients with diabetes and patients with a family history of diabetes [2]. Although dyspnea can result from a hyperglycemia- or hyperinsulinemia-related disease (such as cardiovascular disease, diabetic acidosis, or coronary heart disease [3]), the cause of dyspnea remains unclear.

It has been suggested that T2D is associated with skeletal muscle atrophy. A recent study conducted on a group of patients (18–85 years old) from England showed that T2D is significantly associated with low skeletal muscle mass [4]. Skeletal muscle is the primary site of glucose disposal in response to food intake and insulin, and skeletal muscle atrophy impairs glucose uptake and enhances T2D progression [5]. Respiratory muscle strength has also been shown to be reduced in T2D patients compared to normal control subjects [6], suggesting that T2D can induce muscle atrophy or weakness not only in the skeletal muscle of the extremities but also in respiratory muscles such as the diaphragm and intercostal muscles.

Skeletal muscle is classified depending on muscle fiber characteristics. Type I skeletal muscles are characterized by slow-twitch and oxidative fibers, while type II skeletal muscles are characterized by fast-twitch fibers. Type II fibers are classified further by their metabolic characteristics: type IIa fibers are fatigue-resistant/fast-twitch oxidative fibers, type IIb fibers are fast fatigable/fast-twitch glycolytic fibers, and type IIx fibers have intermediate fatigue resistance between the IIa and IIb fibers [7]. The fiber-type composition differs depending on the location of the skeletal muscle. The gastrocnemius muscle predominantly consists of type IIb muscle fibers [8]. The human diaphragm consists of 50% type I, 25% type IIa, and 25% type IIx muscle fibers. Both the inspiratory and expiratory intercostal muscles have at least 10% more type I fibers than the diaphragm [9]. Slow-twitch (type I) fibers are known to be more insulin-sensitive than fast-twitch (type II) fibers [10]. In accordance, the proportion of slow-twitch fibers in T2D patients is lower than in non-diabetic control subjects [10]. Considering that fiber composition is unique to each muscle, we hypothesize that the mechanism of muscle atrophy or weakening in the skeletal muscles of T2D patients is different from the mechanism of muscle atrophy in the respiratory muscles, which has not been fully studied.

Muscle atrophy results from the imbalance between the synthesis and degradation of proteins [11]. Mammalian/mechanistic target of rapamycin complex 1 (mTORC1) responds to intracellular nutrients and is one of the critical mediators of protein synthesis in muscles [11]. mTOR is a highly conserved serine-threonine kinase that is a master regulator of cell growth. mTORC1 and mTORC2 are biochemically and functionally distinct complexes [5]. mTORC1 responds to changes in amino acids, cellular energy status, oxygen levels, growth factors, and the mechanical contraction of muscles [5]. The muscle-specific loss of mTORC1 reduces muscle mass and oxidative function [12]. Rapamycin, a specific inhibitor of mTOR, targets the function of mTORC1 and dampens postnatal muscle growth and muscle regeneration [13,14]. Sustained mTORC1 activation in muscle has been shown to block both constitutive and starvation-induced autophagy through mTORC1-mediated ULK1 inhibition, leading to late-onset myopathy [15]. Taken together, these previous reports indicate that mTORC1 activity is closely associated with the regulation of muscle mass.

In the present study, we examine the intercostal muscles from *db/db* mice for muscle wasting by comparing these muscles with the gastrocnemius muscle from *db/db* mice. The *db/db* mice carry a mutation in the leptin receptor gene and are a well-established model of obesity-induced type 2 diabetes [16]. mTORC1 activity and Akt phosphorylation levels decreased and were followed by a reduction in FoxO phosphorylation in both the intercostal and gastrocnemius muscles of *db/db* mice. Autophagic flux increased in the gastrocnemius muscle but not in the intercostal muscles of *db/db* mice; however, the levels of Atrogin-1 and MuRF1 increased in both muscle types. Taken together, we conclude that the signaling pathways of muscle wasting in the intercostal muscles of *db/db* mice are different than those in the gastrocnemius muscle of *db/db* mice.

## 2. Results

### 2.1. Both mTORC1 and Akt Are Suppressed in the Intercostal and Gastrocnemius Muscles in db/db Mice

**Figure 1:**
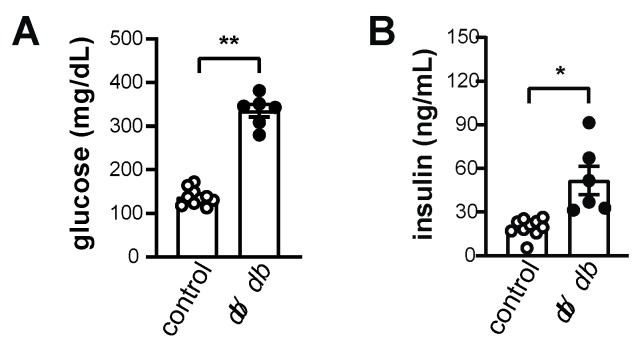
Basal levels of glucose and insulin in *db/db* mice and control mice. (**A**) Basal blood glucose levels in *db/db* and control mice (*n* = 6). (**B**) Basal insulin levels in *db/db* and control mice (*n* = 6). The data are shown as the mean ± standard error of the mean. Statistical analysis was performed with unpaired Student’s *t*-tests. * *p* < 0.05; ** *p* < 0.01; control mice versus *db/db* mice.

First, we compared mTOR signaling of the intercostal muscles to mTOR signaling of the gastrocnemius muscle of *db/db* mice. mTOR is a critical regulator of muscle mass maintenance via its role in controlling the rate of protein synthesis and degradation [17]. Because mTOR is activated by nutrients and growth factors, we isolated muscle samples after a regular meal without overnight fasting to assess the basal level of mTOR activity in the intercostal and gastrocnemius muscles of *db/db* mice. The serum levels of glucose and insulin were relatively high in *db/db* mice compared to non-diabetic control mice (Figure 1A,B), confirming the diabetic status of *db/db* mice that others have reported [18].

**Figure 2:**
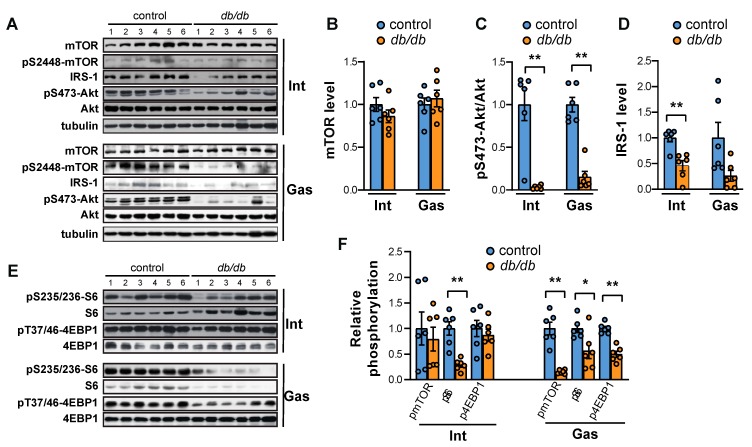
Both mTORC1 and Akt levels are suppressed in both the intercostal and gastrocnemius muscles of *db/db* mice. (**A**) The intercostal and gastrocnemius muscles were lysed and subjected to Western blot analysis (*n* = 6). (**B**–**D**) The relative intensities of the bands were quantified using ImageJ analysis software (*n* = 6). Data are displayed for mTOR compared to tubulin (**B**), pAkt compared to Akt (**C**), and IRS-1 compared to tubulin (**D**). (**E**) The samples were prepared identically to (**A**). (**F**) The relative intensities of the bands were quantified using ImageJ analysis software (*n* = 6). pSer-2448-mTOR compared to mTOR, pSer-235/236-S6 compared to S6, and pThr-37/46-4EBP1 compared to 4EBP1. The data are shown as the mean ± standard error of the mean. Statistics were calculated with unpaired Student’s *t*-tests. * *p* < 0.05; ** *p* < 0.01; control mice versus *db/db* mice. Abbreviations: intercostal muscles (Int); gastrocnemius muscle (Gas).

In contrast, mTOR protein levels remained unchanged in both the intercostal and gastrocnemius muscles of *db/db* mice compared to control mice (Figure 2A,B). The phosphorylation of Akt was significantly decreased in both of the muscle types of *db/db* mice despite the high insulin levels (Figure 2A,C) as previously reported in skeletal muscles [18] and pancreatic β-cells [19]. However, phosphorylation of NDRG1, a downstream target of SGK1 under mTORC2 activity, increased in both the intercostal and gastrocnemius muscles of *db/db* mice (Supplementary Figure S1A,B). This result indicates that the decrease in Akt phosphorylation in both muscle types may be regulated by other factors in addition to mTORC2 activity. Furthermore, IRS-1 expression was completely attenuated in both the intercostal and gastrocnemius muscles from *db/db* mice (Figure 2A,D), offering a molecular explanation for the reduced phosphorylation of Akt through a decrease in phosphoinositide dependent kinase 1 (PDK1). This observation suggests the presence of an insulin signaling defect in the *db/db* mice. The activity of mTORC1 was also decreased in both muscle types, as evidenced by the reduction in phosphorylation of protein S6 at residue Ser-235/236 (Figure 2E,F). However, phosphorylation of Thr-37/46 of 4EBP1 (Figure 2E,F) and Ser-2448 of mTOR (Figure 2A,F), target sites of mTORC1 and S6K1, respectively, was significantly reduced in the gastrocnemius muscle but not the intercostal muscles of *db/db* mice. These results suggest that the overall activity of mTORC1 was diminished in both the intercostal and gastrocnemius muscles but to a lesser extent than in skeletal muscles.

### 2.2. FoxO Activation and the Expression of Atrogin-1 and MuRF1 Are Induced in the Muscles of db/db Mice

**Figure 3:**
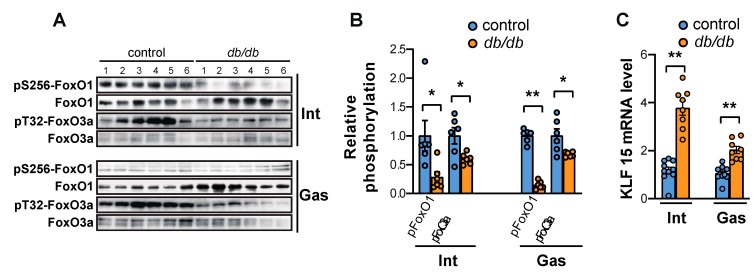
FoxO1 activity, FoxO3a activity, and KLF15 expression are all increased in the intercostal and gastrocnemius muscles of *db/db* mice. (**A**) The intercostal and gastrocnemius muscles were lysed and subjected to Western blot analysis (*n* = 6). (**B**) The relative intensities of the bands were quantified using ImageJ analysis software (*n* = 6). Data are displayed for pSer-256-FoxO1 compared to FoxO1 and pThr-32-FoxO3a compared to FoxO3a. (**C**) The intercostal and gastrocnemius muscles were lysed and subjected to RT-qPCR analysis (*n* = 6). Mouse GAPDH was used to normalize gene expression. The data are shown as the mean ± standard error of the mean. Statistical analysis was performed with unpaired Student’s *t*-tests. * *p* < 0.05; ** *p* < 0.01; control mice versus *db/db* mice. Abbreviations: intercostal muscles (Int); gastrocnemius muscle (Gas).

Akt phosphorylates FoxO transcription factors in response to an increase in insulin receptor (IR) or insulin-like growth factor 1 (IGF-1), resulting in the inhibition of FoxO transcriptional activity, which is one of the main roles of insulin in the liver [19]. Therefore, we assessed the phosphorylation of FoxO1 and FoxO3a in the intercostal and gastrocnemius muscles of *db/db* mice compared to control mice. The phosphorylation of FoxO1 at Ser-256 and FoxO3a at Thr-32 was significantly suppressed in both muscle types tested (Figure 3A,B). Next, we measured the mRNA expression of KLF15, one of several Kruppel-like, zinc finger transcription factors (KLFs) whose expression is directly induced by glucocorticoids [20]. KLF15 mRNA levels were increased significantly in the intercostal muscles of *db/db* mice, whereas they were only mildly enhanced in the gastrocnemius muscle of *db/db* mice (Figure 3C).

**Figure 4:**
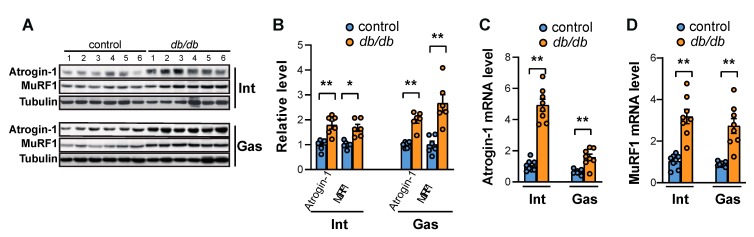
The levels of Atrogin-1 and MuRF1 are increased in the intercostal and gastrocnemius muscles of *db/db* mice. (**A**) The intercostal and gastrocnemius muscles were lysed and subjected to Western blot analysis (*n* = 6). (**B**) The relative intensities of the bands were quantified using ImageJ analysis software (*n* = 6). Atrogin-1 and MuRF1 levels are shown, both in comparison with tubulin levels. (**C**,**D**) The intercostal and gastrocnemius muscles were lysed and subjected to RT-qPCR analysis (*n* = 6). Mouse GAPDH was used to normalize gene expression. The data are shown as the mean ± standard error of the mean. Statistical analysis was performed with unpaired Student’s *t*-tests. * *p* < 0.05; ** *p* < 0.01; control mice versus *db/db* mice. Abbreviations: intercostal muscles (Int); gastrocnemius muscle (Gas).

FoxO transcription factors are known to regulate a wide range of atrophy-related genes in muscle tissue, including Atrogin-1, MuRF1, and autophagy genes [21,22]. KLF15 is also a well-known transcription factor that regulates the expression of Atrogin-1 and MuRF1 [20]. Atrogin-1 and MuRF1 are E3 ubiquitin ligases that are expressed in skeletal muscle and bind to polyubiquitinated proteins to direct them for subsequent degradation by the 26S proteasome [23]. The expression levels of Atrogin-1 and MuRF1 were increased in the gastrocnemius muscle (as previously reported [24]), as well as in the intercostal muscles of *db/db* mice (Figure 4A,B), indicating that the increase in expression of Atrogin-1 and MuRF1 is correlated with FoxO activation and KLF15 expression in both the intercostal and gastrocnemius muscles of insulin-resistant *db/db* mice. Consistent with changes in protein expression, mRNA expression levels of Atrogin-1 (Figure 4C) and MuRF1 (Figure 4D) were significantly increased in the intercostal and gastrocnemius muscles of *db/db* mice compared to control mice. These results suggested that ubiquitin-dependent protein degradation was activated in both the intercostal and gastrocnemius muscles of *db/db* mice.

### 2.3. Autophagy is Elicited in the Gastrocnemius Muscle but not the Intercostal Muscles of db/db Mice

**Figure 5:**
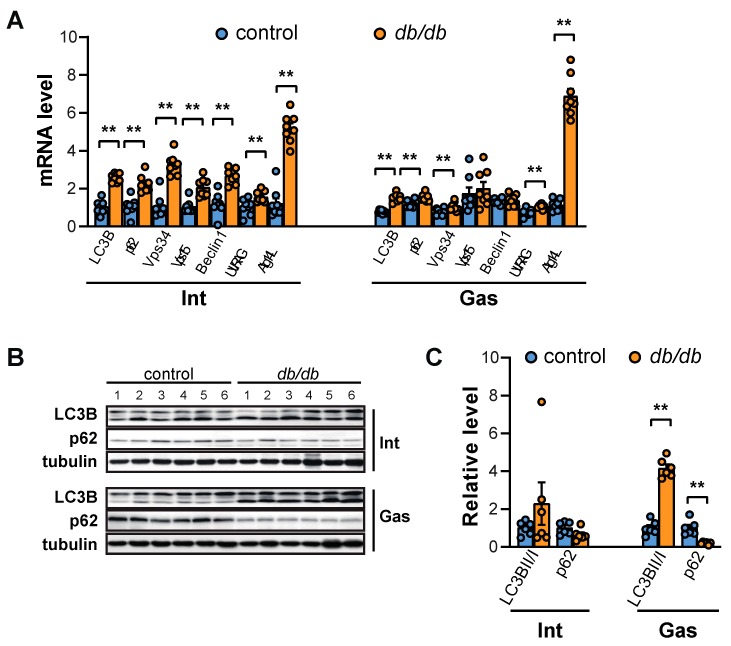
Autophagic flux is increased in the gastrocnemius muscle but not in the intercostal muscles of *db/db* mice. (**A**) The intercostal and gastrocnemius muscles were lysed and subjected to RT-qPCR analysis (*n* = 6). Mouse GAPDH was used to normalize gene expression. (**B**) The intercostal and gastrocnemius muscles were lysed and subjected to Western blot analysis (*n* = 6). (**C**) The relative intensities of the bands were quantified using ImageJ analysis software (*n* = 6). Data are presented for LC3BII compared to LC3BI and p62 compared to tubulin. The data are shown as the mean ± standard error of the mean. Statistical analysis was performed with unpaired Student’s *t*-tests. ** *p* < 0.01; control mice versus *db/db* mice. Abbreviations: intercostal muscles (Int); gastrocnemius muscle (Gas).

FoxO stimulates lysosomal proteolysis in muscle cells by activating the expression of autophagy-related genes [22]. The mRNA levels of autophagosome-specific phosphatidyl inositol 3-kinase (PI3K) complex-related genes (Vps34, Vps15, Beclin1, UVRAG, and Atg14L) and autophagosome-located genes (LC3B and p62) were significantly increased in the intercostal muscles of *db/db* mice (Figure 5A). However, only Vps34, UVRAG, Atg14L, LC3B, and p62 mRNA levels were notably enhanced in the gastrocnemius muscle of *db/db* mice (Figure 5A). In addition, autophagic flux, characterized by an increase in LC3BII/I and a decrease in p62 protein levels, was enhanced in the gastrocnemius muscle but remained unchanged in intercostal muscles (Figure 5B,C). Next, we examined autophagic flux in the muscles of diet-induced obese (DIO) mice to determine whether the increase in autophagic flux in the gastrocnemius muscles was induced in other T2D mouse models. Exposure to high-fat diets (HFDs) often induces the development of T2D and obesity [25]. Serum glucose levels were significantly elevated (up to 1.6-fold) in HFD-fed mice compared to mice fed regular chow (RC) diets (Supplementary Figure S2A), but glucose levels were generally lower than those of *db/db* mice (Figure 1A) as previously reported [26]. Akt phosphorylation at Ser473 was increased in both the intercostal and gastrocnemius muscles (Supplementary Figure S2B,C). Notably, the autophagic flux in both muscles of HFD-mice, indicated by p62 and LC3BII/I levels, was comparable to that in both muscles of RC-mice (Supplementary Figure S2D,E). Although *db/db* mice and DIO mice are used as models of obesity and T2D, DIO mice showed only mild hyperglycemia (Supplementary Figure S2A), which may not have been enough to result in the signaling events observed in muscles of *db/db* mice. Hence, these results suggest that a more severe diabetic condition could be a prerequisite for the differential increase in autophagic flux observed in the gastrocnemius muscle of *db/db* mice.

**Figure 6:**
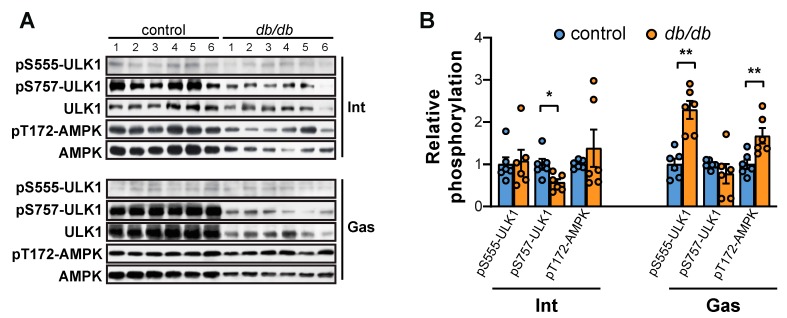
The initiation of autophagy is induced in the gastrocnemius muscle of *db/db* mice through phosphorylation of Ser-555 in ULK1 and Thr-172 in AMPK. (**A**) The intercostal and gastrocnemius muscles were lysed and subjected to Western blot analysis (*n* = 6). (**B**) The relative intensities of the bands were quantified using ImageJ analysis software (*n* = 6). Data are presented for pSer-555-ULK1 compared to ULK1, pSer-757-ULK1 compared to ULK1, and pThr-172-AMPK compared to AMPK. The data are shown as the mean ± standard error of the mean. Statistics analysis was performed with unpaired Student’s *t*-tests. * *p* < 0.05; ** *p* < 0.01; control mice versus *db/db* mice. Abbreviations: intercostal muscles (Int); gastrocnemius muscle (Gas).

Consistent with this observation, the induction of autophagosome machinery, demonstrated by phosphorylation of AMP-activated protein kinase (AMPK) at Thr-172 and ULK1 at Ser-555, increased in the gastrocnemius muscle of *db/db* mice but not in the intercostal muscles (Figure 6A,B). The phosphorylation of ULK1 at Ser-757, a regulatory site of mTORC1, was significantly decreased in the intercostal muscles, reflecting the reduced activity of mTORC1 (Figure 6A,B). Together, these results suggest that autophagy is induced in the gastrocnemius muscle but not in the intercostal muscles of *db/db* mice.

## 3. Discussion

The association between diabetes and a decrease in skeletal muscle mass and muscle strength has been well studied in the past. In previous studies, diabetic patients showed an annual loss of muscle mass and muscle strength of up to 26% and 33%, respectively, compared to normal control subjects [27,28,29,30]. The mechanism of how muscle atrophy is aggravated by diabetes has been studied using animal models and human samples. Insulin deficiency in streptozotocin-induced type 1 diabetic (T1D) rats induces significant muscle atrophy that leads to increased proteasomal protein degradation in muscle cells [31]. Compared to the T1D mouse model, muscle atrophy in T2D animal models is more complicated. Muscle mass remains unchanged or shows only a mild decrease in the high fat diet-induced T2D mouse model [32,33,34], whereas muscle size is severely reduced in the *db/db* T2D mouse model [35,36]. Muscle atrophy of T2D could be more important than muscle atrophy of T1D from a clinical perspective because the incidence rate of T2D is 90–95% of all diabetes cases [31]. However, it is unclear whether muscle loss is determined by a defect in insulin signaling or the degree or elevated insulin and glucose.

**Figure 7:**
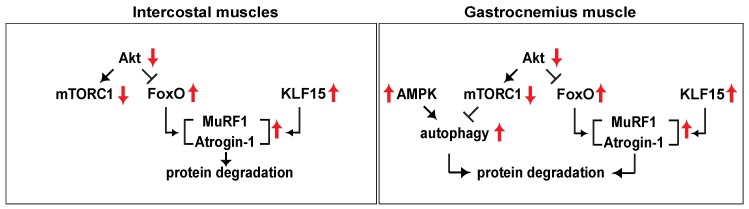
Proposed model of protein degradation signaling events in the intercostal and the gastrocnemius muscles of *db/db* mice. Akt phosphorylation and mTORC1 activity decrease; subsequently, FoxO activity and KLF15 expression increase leading to an increase in Atrogin-1 and MuRF1 expression. The increase in AMPK phosphorylation at Thr-172 and subsequent ULK1 phosphorylation at Ser-555 is accompanied by the enhancement in autophagic flux in the gastrocnemius muscles but not in the intercostal muscles of *db/db* mice.

It has been theorized that muscle atrophy occurs in the respiratory muscles in patients with diabetic to the same extent as that in the extremity muscles. The decrease in respiratory muscle strength in patients with T2D is correlated with an increase in the incidence of aspiration, aspiration pneumonia, physical functional limitations, and mortality [37]. Furthermore, pneumonia can cause death in patients with T2D, especially older patients [38]; a Japanese study found that 12% of diabetic patient deaths were due to pneumonia [38]. Therefore, a comparison of muscle atrophy and its associated mechanisms between the extremity and respiratory muscles of T2D patients is warranted. In the present study, we analyzed the signaling pathways of muscle loss in the intercostal muscles and the gastrocnemius muscles of *db/db* mice. Notably, we found that the expression levels of MuRF1 and Atrogin-1 were increased in both the intercostal and gastrocnemius muscles of *db/db* mice, whereas autophagic flux was increased in the gastrocnemius muscle but not the intercostal muscles (Figure 7). These results suggest that the mechanism of muscle wasting in the intercostal muscles is different from that in the gastrocnemius muscle.

Considering the low basal activity of mTORC1 in the muscles of *db/db* mice and the critical role that mTORC1 plays in protein synthesis, we speculate that a decrease in protein synthesis could be the cause of the observed reduction in muscle mass. In addition, an increase in the phosphorylation of AMPK (Figure 5) and Atrogin-1 expression (Figure 4) could further impair muscle protein synthesis. An increase in the phosphorylation of AMPK inhibits mTORC1 activity by phosphorylating TSC2 [39] and raptor [40]. Atrogin-1 reduces protein synthesis by stimulating the degradation of eIF3f, an essential factor controlling protein translation [41]. In the current study, we observed that an increase in the phosphorylation of AMPK was significant in the gastrocnemius muscle of *db/db* mice but not in the intercostal muscles. This observation suggests that the interruption of protein synthesis by activated AMPK may not regulate intercostal muscle mass. Protein half-life was determined by basal protein breakdown, which affects the total protein content of the cell [42], suggesting that a decrease in protein synthesis alone might not evoke muscle atrophy. Whether protein synthesis or protein degradation is more important for maintaining intercostal muscle mass warrants further study.

An increase in the expression levels of Atrogin-1 and MuRF1 is responsible for shifting the net maintenance of protein levels towards protein degradation during conditions that induce muscle atrophy [43]. In the present study, impaired IRS-1 expression and Akt phosphorylation led to reduced phosphorylation of FoxO transcription factors, which can translocate into the nucleus and induce gene expression. Atrogin-1 and MuRF1 dramatically increase during a catabolic state under FoxO regulation [44] and glucocorticoid-induced KLF15 expression [20]. FoxO family transcription factors have been shown to bind to the promoters of Atrogin-1 and MuRF1 [45]. In addition, KLF15 can give rise to Atrogin-1 and MuRF1 directly, as well as indirectly, through FoxO expression, because KLF15 also induces FoxO1 and FoxO3a. The significant increase in expressions of Atrogin-1 and MuRF1 (Figure 4) was accompanied by the activation of FoxO transcription factors and KLF15 in both the intercostal and gastrocnemius muscles of *db/db* mice (Figure 3). Notably, we showed that muscle wasting in the intercostal muscles is different from that in the gastrocnemius muscle of *db/db* mice; autophagic flux was induced only in the gastrocnemius muscle but not in the intercostal muscles, whereas Atrogin-1 and MuRF1, two ubiquitin ligases that are known to be present in skeletal muscles, were enhanced in both muscles (Figure 5). These results suggest that another regulatory mechanism may block autophagic flux in the intercostal muscles, resulting in only mild protein degradation under diabetic conditions and the relative preservation of muscle mass in the intercostal muscles.

The role of autophagy in skeletal muscles is complex. An increase in autophagic flux is observed under conditions that induce muscle atrophy (such as bed rest, cachexia, or denervation), but the inhibition of autophagy also induces muscle atrophy via the accumulation of damaged proteins [42]. In the current study, autophagic flux in the gastrocnemius muscle of *db/db* mice increased and was accompanied by an increase in phosphorylation of ULK1 and AMPK (Figure 6). ULK1 is required for autophagy initiation [46]. ULK1 activation is induced by the loss of mTOR-induced Ser-757 phosphorylation and the addition of AMPK-induced Ser-555 phosphorylation [46]. Considering that phosphorylation of ULK1 at Ser-555 and AMPK at Thr-172 remained unchanged in the intercostal muscles of *db/db* mice, the decrease in ULK1 Ser-757 phosphorylation may not have been sufficient to activate ULK1 and the subsequent autophagy-inducing signal, suggesting that the role of AMPK in autophagy initiation is more critical than the role of mTOR. In addition to the direct ULK1 activation and mTOR inhibition, AMPK regulates autophagy in diverse ways. AMPK activates FoxO3a, which upregulates the transcription of muscle-specific atrogenes [47]. AMPK also phosphorylates Beclin1, which can activate the pro-autophagic Vps34 complex or the non-autophagic Vps34 complex [48]. In *db/db* mice, AMPK was significantly activated in the gastrocnemius muscle but not in the intercostal muscles, subsequently leading to an increase in autophagic flux, which supports the important role of AMPK in the autophagy of muscles. In addition, the upregulation of LC3B and p62 at the mRNA level (Figure 5A) did not correspond to the changes in their protein levels in the current study (Figure 5B,C). Since these proteins are sequestered in the autophagosome and destroyed when the autophagosome and lysosome fuse, transcriptional upregulation of the mRNA of these genes represents an increase in the expression of autophagy-related genes consistent with an increase in autophagic flux (Figure 5B,C) [42].

Although this is the first report to illustrate the differences in the molecular signaling in muscle protein synthesis and degradation between the intercostal and gastrocnemius muscles under diabetic conditions in addition to the presence of muscle protein wasting signaling events in the intercostal muscles of *db/db* mice, several limitations restrict the interpretation of the current study. Because the differential increases in autophagic flux in the gastrocnemius muscles of *db/db* mice were not observed in DIO mice (Supplementary Figure S2D,E), the molecular events that we observed in *db/db* mice are limited to *db/db* mice-like conditions. Hence, we speculate that the differences between the intercostal and the gastrocnemius muscles only become obvious with severe diabetic symptoms (e.g., high glucose levels, high insulin levels, severe weight gain), as seen in *db/db* mice. Severe diabetic conditions could be required to result in the differences observed between the intercostal muscles and the gastrocnemius muscle. Another limitation of the current study is that we primarily focused on differentiating the molecular events in the intercostal muscles from the molecular events in the gastrocnemius muscle. The mechanistic basis of the signaling of muscle protein synthesis and degradation in the intercostal and gastrocnemius muscles remains to be addressed.

The present study suggests that the increase in Atrogin-1 and MuRF1 expression in the intercostal muscles may result in intercostal muscle wasting in *db/db* mice (Figure 7). Moreover, we propose that the muscle loss caused by T2D in the intercostal muscles is different from gastrocnemius muscle loss, suggesting that each muscle is regulated differently in T2D (Figure 7). It is plausible that the respiratory muscles tend to preserve muscle mass under T2D conditions considering that autophagic flux was not induced in these muscles. It was reported that the incidence of T2D in chronic obstructive pulmonary disease (COPD) patients is high [49], and a modest association between patients with moderate COPD and patients with an increased risk of diabetes has been reported [50]. Therefore, the increase in muscle wasting observed in T2D patients may be associated with mortality caused by respiration difficulties; this hypothesis will need to be studied further to analyze the mechanism of muscle atrophy in the intercostal muscles and to provide a clearer understanding of the clinical relevance of respiratory muscle maintenance.

## 4. Materials and Methods

### 4.1. Antibodies and Other Reagents

Antibodies were purchased from the following sources: LC3B from Novus Biologicals (Centennial, CO, USA); tubulin from Abcam (Cambridge, UK); MuRF1 and Atrogin-1 from Santa Cruz Biotechnology (Dallas, TX, USA); pT32-Foxo3a from Millipore (Burlington, MA, USA); secondary antibodies from Jackson ImmunoResearch Laboratories, Inc. (West Grove, PA, USA). All other primary antibodies were obtained from Cell Signaling Technology (Danvers, MA, USA). All other reagents were purchased from Sigma-Aldrich (St. Louis, MO, USA).

### 4.2. Animals

Thirteen-week-old BKS(D)-*Lepr^db^*/JOrlRj (*db/db*) male mice (Jackson Laboratory, Bar Harbor, ME, USA) and thirteen-week-old C57BL/6J male mice (Daehan Bio Link, Chungbuk, Repubulic of Korea) were kept in temperature-controlled cages (20–22 °C) with a reverse light-dark cycle (8 pm–8 am) with free access to water and food. All experimental protocols used for animal studies, as well as their maintenance and care, were conducted in accordance with Gachon University Animal Care Guidelines. All animal procedures were approved by the Gachon University of Medicine and the Institutional Animal Care and Use Committee (IACUC) (permission number: LCDI-2017-0121, 1 October 2017).

### 4.3. Tissue Lysis, Immunoprecipitation, and Western Blot Analysis

Total protein samples were extracted from muscle using T-PER tissue protein extraction reagent supplemented with protease and phosphatase inhibitors (Thermo Scientific, Waltham, MA, USA). The muscle samples were disrupted with a steel bead in the extraction buffer using a TissueLyser II (Qiagen, Hilden, Germany) set at 30 Hz for 1–2 min. The supernatant was collected after microcentrifugation at 13,000× *g* for 10 min at 4 °C and then boiled in sodium dodecyl sulfate (SDS) sample buffer for 5 min. Proteins were resolved by electrophoresis on SDS-polyacrylamide gels and then transferred to polyvinylidene fluoride membranes (Millipore, Burlington, MA, USA). Antibody incubations were followed according to the manufacturer’s recommended conditions. Immobilon Western Chemiluminescent HRP substrate (Millipore, Burlington, MA, USA) was used to detect horseradish peroxidase-conjugated secondary antibodies. Western blot band intensities were quantified by densitometry of x-ray film images using the software ImageJ.

### 4.4. RNA Isolation and Real-Time Quantitative Reverse Transcription Polymerase Chain Reaction (RT-qPCR)

The muscles were lysed in TRIzol reagent (Thermo Fisher Scientific, Waltham, MA, USA) using a TissueLyser II (Qiagen, Hilden, Germany) set at 30 Hz for 30 s, 6–8 times. Total RNA was isolated according to the manufacturer’s protocol. cDNA was synthesized from 2 μg of RNA using the TOPscript^TM^ RT DryMIX kit (dT18 plus) (Enzynomics, Daejeon, Repubulic of Korea). Real-time quantitative PCR analyses were performed using a CFX384 C1000 thermal cycler (Bio-Rad, Hercules, CA, USA) using TOPreal^TM^ qPCR 2X PreMIX (SYBR Green with high ROX) (Enzynomics, Daejeon, Repubulic of Korea). For normalization of gene expression, mouse glyceraldehyde 3-phosphate dehydrogenase (GAPDH) was used. All primers used for RT-qPCR are shown in Table 1. 

### 4.5. Statistical Analysis

All data are presented as the mean ± standard error of the mean (SEM). Where necessary, statistical significance was determined by performing a Student’s *t*-test. *p*-Values < 0.05 were considered statistically significant.

## Figures and Tables

**Table 1:** Primers used for RT-qPCR analyses.

Target Gene	Sequence of Primer
*mouse GAPDH F*	*TCCCACTCTTCCACCTTCGA*
*mouse GAPDH R*	*CAGGAAATGAGCTTGACAAAGTTG*
*mouse p62 F*	*GAAGCTGCCCTATACCCACA*
*mouse p62 R*	*GAGAAACCCATGGACAGCAT*
*mouse LC3B F*	*TTATAGAGCGATACAAGGGGGAG*
*mouse LC3B R*	*CGCCGTCTGATTATCTTGATGAG*
*mouse Vps34 F*	*CCTGGACATCAACGTGCAG*
*mouse Vps34 R*	*TGTCTCTTGGTATAGCCCAGAAA*
*mouse Vps15 F*	*GGTGGTCACGTTGCTAAGC*
*mouse Vps15 R*	*CGCAGGTGCCAATCATTCTTAT*
*mouse Beclin1 F*	*ATGGAGGGGTCTAAGGCGTC*
*mouse Beclin1 R*	*TCCTCTCCTGAGTTAGCCTCT*
*mouse UVRAG F*	*ACATCGCTGCTCGGAACATT*
*mouse UVRAG R*	*CTCCACGTCGGATTCAAGGAA*
*mouse Atg14L F*	*GAGGGCCTTTACGTGGCTG*
*mouse Atg14L R*	*AATAGACGAAATCACCGCTCTG*
*mouse MuRF1 F*	*TCCTGGACGAGAAGAAGAGC*
*mouse MuRF1 R*	*TGCTCCCTGTACTGGAGGAT*
*mouse Atrogin-1 F*	*GCAACAAGGAGGTATACAGTAAGG*
*mouse Atrogin-1 R*	*TCCTTCGTACTTCCTTTGTGAAC*
*mouse KLF15 F*	*CTGCAGCAAGATGTACACCAA*
*mouse KLF15 R*	*TCATCTGAGCGTGAAAACCTC*

## Supplementary Materials

Supplementary materials can be found at https://www.mdpi.com/1422-0067/20/23/6062/s1. Figure S1: The phosphorylation of NDRG1 did not change significantly in either the intercostal or gastrocnemius muscles of *db/db* mice. Figure S2: Akt phosphorylation and autophagic flux did not change in high fat diet-fed mice.

## Abbreviations

mTOR	Mammalian target of rapamycin
T1D	Type 1 diabetes
T2D	Type 2 diabetes
mTORC1	mTOR complex1
mTORC2	mTOR complex2
*db/db* mice	Diabetic mice
DIO mice	Diet-induced obese mice
HFD	High fat diet
RC	Regular Chow

## References

[1] Martin-Timon I., Sevillano-Collantes C., Segura-Galindo A., Del Canizo-Gomez F.J. (2014). Type 2 diabetes and cardiovascular disease: Have all risk factors the same strength?. World J. Diabetes.

[2] Zellweger M.J., Hachamovitch R., Kang X., Hayes S.W., Friedman J.D., Germano G., Pfisterer M.E., Berman D.S. (2004). Prognostic relevance of symptoms versus objective evidence of coronary artery disease in diabetic patients. Eur. Heart J..

[3] Murthy V.L., Naya M., Foster C.R., Gaber M., Hainer J., Klein J., Dorbala S., Blankstein R., Carli M.F.D. (2012). Association Between Coronary Vascular Dysfunction and Cardiac Mortality in Patients With and Without Diabetes Mellitus. Circulation.

[4] Han T.S., Al-Gindan Y.Y., Govan L., Hankey C.R., Lean M.E.J. (2019). Associations of BMI, waist circumference, body fat, and skeletal muscle with type 2 diabetes in adults. Acta Diabetol..

[5] Laplante M., Sabatini D.M. (2012). mTOR signaling in growth control and disease. Cell.

[6] Van Eetvelde B.L.M., Cambier D., Vanden Wyngaert K., Celie B., Calders P. (2018). The Influence of Clinically Diagnosed Neuropathy on Respiratory Muscle Strength in Type 2 Diabetes Mellitus. J. Diabetes Res..

[7] Schiaffino S., Reggiani C. (2011). Fiber types in mammalian skeletal muscles. Physiol. Rev..

[8] Hamalainen N., Pette D. (1993). The histochemical profiles of fast fiber types IIB, IID, and IIA in skeletal muscles of mouse, rat, and rabbit. J. Histochem. Cytochem..

[9] Mizuno M. (1991). Human respiratory muscles: Fibre morphology and capillary supply. Eur. Respir. J..

[10] Gaster M., Staehr P., Beck-Nielsen H., Schroder H.D., Handberg A. (2001). GLUT4 Is Reduced in Slow Muscle Fibers of Type 2 Diabetic Patients. Is Insulin Resistance in Type 2 Diabetes a Slow, Type 1 Fiber Disease?. Diabetes.

[11] Schiaffino S., Dyar K.A., Ciciliot S., Blaauw B., Sandri M. (2013). Mechanisms regulating skeletal muscle growth and atrophy. FEBS J..

[12] Bentzinger C.F., Romanino K., Cloetta D., Lin S., Mascarenhas J.B., Oliveri F., Xia J., Casanova E., Costa C.F., Brink M. (2008). Skeletal muscle-Specific ablation of raptor, but not of rictor, causes metabolic changes and results in muscle dystrophy. Cell Metab..

[13] Pallafacchina G., Calabria E., Serrano A.L., Kalhovde J.M., Schiaffino S. (2002). A protein kinase B-Dependent and rapamycin-Sensitive pathway controls skeletal muscle growth but not fiber type specification. Proc. Natl. Acad. Sci. USA.

[14] Ge Y., Wu A.L., Warnes C., Liu J., Zhang C., Kawasome H., Terada N., Boppart M.D., Schoenherr C.J., Chen J. (2009). mTOR regulates skeletal muscle regeneration in vivo through kinase-dependent and kinase-Independent mechanisms. Am. J. Physiol. Cell Physiol..

[15] Castets P., Lin S., Rion N., Di Fulvio S., Romanino K., Guridi M., Frank S., Tintignac L.A., Sinnreich M., Ruegg M.A. (2013). Sustained activation of mTORC1 in skeletal muscle inhibits constitutive and starvation-induced autophagy and causes a severe, late-Onset myopathy. Cell Metab..

[16] Bogdanov P., Corraliza L., Villena J.A., Carvalho A.R., Garcia-Arumi J., Ramos D., Ruberte J., Simo R., Hernandez C. (2014). The db/db mouse: A useful model for the study of diabetic retinal neurodegeneration. PLoS ONE.

[17] Tang H., Inoki K., Brooks S.V., Okazawa H., Lee M., Wang J., Kim M., Kennedy C.L., Macpherson P.C.D., Ji X. (2019). mTORC1 underlies age-Related muscle fiber damage and loss by inducing oxidative stress and catabolism. Aging Cell.

[18] Anil T.M., Harish C., Lakshmi M.N., Harsha K., Onkaramurthy M., Sathish Kumar V., Shree N., Geetha V., Balamurali G.V., Gopala A.S. (2014). CNX-012-570, a direct AMPK activator provides strong glycemic and lipid control along with significant reduction in body weight; studies from both diet-Induced obese mice and db/db mice models. Cardiovasc. Diabetol..

[19] O’Neill B.T., Bhardwaj G., Penniman C.M., Krumpoch M.T., Suarez Beltran P.A., Klaus K., Poro K., Li M., Pan H., Dreyfuss J.M. (2018). FoxO Transcription Factors are Critical Regulators of Diabetes-Related Muscle Atrophy. Diabetes.

[20] Shimizu N., Yoshikawa N., Ito N., Maruyama T., Suzuki Y., Takeda S., Nakae J., Tagata Y., Nishitani S., Takehana K. (2011). Crosstalk between glucocorticoid receptor and nutritional sensor mTOR in skeletal muscle. Cell Metab..

[21] Sandri M., Sandri C., Gilbert A., Skurk C., Calabria E., Picard A., Walsh K., Schiaffino S., Lecker S.H., Goldberg A.L. (2004). Foxo transcription factors induce the atrophy-Related ubiquitin ligase atrogin-1 and cause skeletal muscle atrophy. Cell.

[22] Zhao J., Brault J.J., Schild A., Cao P., Sandri M., Schiaffino S., Lecker S.H., Goldberg A.L. (2007). FoxO3 coordinately activates protein degradation by the autophagic/lysosomal and proteasomal pathways in atrophying muscle cells. Cell Metab..

[23] Gumucio J.P., Mendias C.L. (2013). Atrogin-1, MuRF-1, and sarcopenia. Endocrine.

[24] Wang H., Liu D., Cao P., Lecker S., Hu Z. (2010). Atrogin-1 Affects Muscle Protein Synthesis and Degradation When Energy Metabolism Is Impaired by the Antidiabetes Drug Berberine. Diabetes.

[25] Lutz T.A., Woods S.C. (2012). Overview of animal models of obesity. Curr. Protoc. Pharmacol..

[26] Rong J.X., Qiu Y., Hansen M.K., Zhu L., Zhang V., Xie M., Okamoto Y., Mattie M.D., Higashiyama H., Asano S. (2007). Adipose mitochondrial biogenesis is suppressed in db/db and high-Fat diet-Fed mice and improved by rosiglitazone. Diabetes.

[27] Goodpaster B.H., Park S.W., Harris T.B., Kritchevsky S.B., Nevitt M., Schwartz A.V., Simonsick E.M., Tylavsky F.A., Visser M., Newman A.B. (2006). The loss of skeletal muscle strength, mass, and quality in older adults: The health, aging and body composition study. J. Gerontol. A Biol. Sci. Med. Sci..

[28] Kim T.N., Park M.S., Yang S.J., Yoo H.J., Kang H.J., Song W., Seo J.A., Kim S.G., Kim N.H., Baik S.H. (2010). Prevalence and determinant factors of sarcopenia in patients with type 2 diabetes: The Korean Sarcopenic Obesity Study (KSOS). Diabetes Care.

[29] Park S.W., Goodpaster B.H., Lee J.S., Kuller L.H., Boudreau R., De Rekeneire N., Harris T.B., Kritchevsky S., Tylavsky F.A., Nevitt M. (2009). Excessive loss of skeletal muscle mass in older adults with type 2 diabetes. Diabetes Care.

[30] Park S.W., Goodpaster B.H., Strotmeyer E.S., Kuller L.H., Broudeau R., Kammerer C., De Rekeneire N., Harris T.B., Schwartz A.V., Tylavsky F.A. (2007). Accelerated loss of skeletal muscle strength in older adults with type 2 diabetes: The health, aging, and body composition study. Diabetes Care.

[31] Pepato M.T., Migliorini R.H., Goldberg A.L., Kettelhut I.C. (1996). Role of different proteolytic pathways in degradation of muscle protein from streptozotocin-Diabetic rats. Am. J. Physiol..

[32] Sitnick M., Bodine S.C., Rutledge J.C. (2009). Chronic high fat feeding attenuates load-Induced hypertrophy in mice. J. Physiol..

[33] Turpin S.M., Ryall J.G., Southgate R., Darby I., Hevener A.L., Febbraio M.A., Kemp B.E., Lynch G.S., Watt M.J. (2009). Examination of ‘lipotoxicity’ in skeletal muscle of high-Fat fed and ob/ob mice. J. Physiol..

[34] Yokota T., Kinugawa S., Hirabayashi K., Matsushima S., Inoue N., Ohta Y., Hamaguchi S., Sobirin M.A., Ono T., Suga T. (2009). Oxidative stress in skeletal muscle impairs mitochondrial respiration and limits exercise capacity in type 2 diabetic mice. Am. J. Physiol. Heart Circ. Physiol..

[35] Wang X., Hu Z., Hu J., Du J., Mitch W.E. (2006). Insulin resistance accelerates muscle protein degradation: Activation of the ubiquitin-proteasome pathway by defects in muscle cell signaling. Endocrinology.

[36] Ostler J.E., Maurya S.K., Dials J., Roof S.R., Devor S.T., Ziolo M.T., Periasamy M. (2014). Effects of insulin resistance on skeletal muscle growth and exercise capacity in type 2 diabetic mouse models. Am. J. Physiol. Endocrinol. Metab..

[37] Kera T., Kawai H., Hirano H., Kojima M., Watanabe Y., Motokawa K., Fujiwara Y., Ihara K., Kim H., Obuchi S. (2019). Definition of Respiratory Sarcopenia With Peak Expiratory Flow Rate. J. Am. Med. Dir. Assoc..

[38] Nakamura J., Kamiya H., Haneda M., Inagaki N., Tanizawa Y., Araki E., Ueki K., Nakayama T. (2017). Causes of death in Japanese patients with diabetes based on the results of a survey of 45,708 cases during 2001–2010: Report of the Committee on Causes of Death in Diabetes Mellitus. J. Diabetes Investig..

[39] Inoki K., Zhu T., Guan K.L. (2003). TSC2 mediates cellular energy response to control cell growth and survival. Cell.

[40] Gwinn D.M., Shackelford D.B., Egan D.F., Mihaylova M.M., Mery A., Vasquez D.S., Turk B.E., Shaw R.J. (2008). AMPK phosphorylation of raptor mediates a metabolic checkpoint. Mol. Cell.

[41] Lagirand-Cantaloube J., Offner N., Csibi A., Leibovitch M.P., Batonnet-Pichon S., Tintignac L.A., Segura C.T., Leibovitch S.A. (2008). The initiation factor eIF3-F is a major target for atrogin1/MAFbx function in skeletal muscle atrophy. Embo. J..

[42] Sandri M. (2013). Protein breakdown in muscle wasting: Role of autophagy-Lysosome and ubiquitin-Proteasome. Int. J. Biochem. Cell Biol..

[43] Bodine S.C., Baehr L.M. (2014). Skeletal muscle atrophy and the E3 ubiquitin ligases MuRF1 and MAFbx/atrogin-1. Am. J. Physiol. Endocrinol. Metab..

[44] Lecker S.H., Goldberg A.L., Mitch W.E. (2006). Protein Degradation by the Ubiquitin–Proteasome Pathway in Normal and Disease States. J. Soc. Nephrol..

[45] Waddell D.S., Baehr L.M., Van Den Brandt J., Johnsen S.A., Reichardt H.M., Furlow J.D., Bodine S.C. (2008). The glucocorticoid receptor and FOXO1 synergistically activate the skeletal muscle atrophy-Associated MuRF1 gene. Am. J. Physiol. Endocrinol. Metab..

[46] Egan Daniel F., Chun Matthew G.H., Vamos M., Zou H., Rong J., Miller Chad J., Lou Hua J., Raveendra-Panickar D., Yang C.C., Sheffler Douglas J. (2015). Small Molecule Inhibition of the Autophagy Kinase ULK1 and Identification of ULK1 Substrates. Mol. Cell.

[47] Sanchez A.M., Csibi A., Raibon A., Cornille K., Gay S., Bernardi H., Candau R. (2012). AMPK promotes skeletal muscle autophagy through activation of forkhead FoxO3a and interaction with Ulk1. J. Cell Biochem..

[48] Kim J., Kim Y.C., Fang C., Russell R.C., Kim J.H., Fan W., Liu R., Zhong Q., Guan K.L. (2013). Differential regulation of distinct Vps34 complexes by AMPK in nutrient stress and autophagy. Cell.

[49] Gayle A., Dickinson S., Poole C., Pang M., Fauconnot O., Quint J.K. (2019). Incidence of type II diabetes in chronic obstructive pulmonary disease: A nested case-Control study. NPJ Prim. Care Respir. Med..

[50] Mannino D.M., Thorn D., Swensen A., Holguin F. (2008). Prevalence and outcomes of diabetes, hypertension and cardiovascular disease in COPD. Eur. Respir. J..

