# Supplementary material for: Analysis of the Molecular Signaling Signatures of Muscle Protein Wasting Between the Intercostal Muscles and the Gastrocnemius Muscles in db/db Mice
Source: Int J Mol Sci. 2019 Dec 1;20(23):6062. doi: 10.3390/ijms20236062 (PMC6929000; doi:10.3390/ijms20236062)
Supplement: Supplementary file 1 [file ijms-20-06062-s001.pdf]

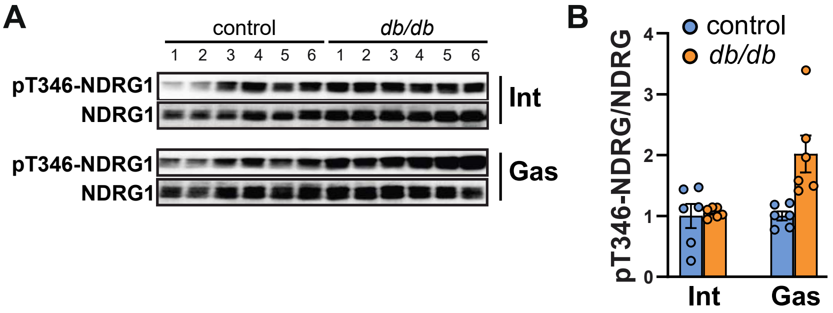

Supplementary Figure S1. The phosphorylation of NDRG1 did not change significantly in either the intercostal or gastrocnemius muscles of *db/db* mice. (A) The intercostal and gastrocnemius muscles were lysed and subjected to western blot analysis (n=6). (B) The relative intensities of the bands were quantified using ImageJ analysis software (n=6). Data are displayed for pThr-346-NDRG1 compared to NDRG1. The data are shown as the mean ± standard error of the mean. Abbreviations: intercostal muscles (Int); gastrocnemius muscle (Gas).

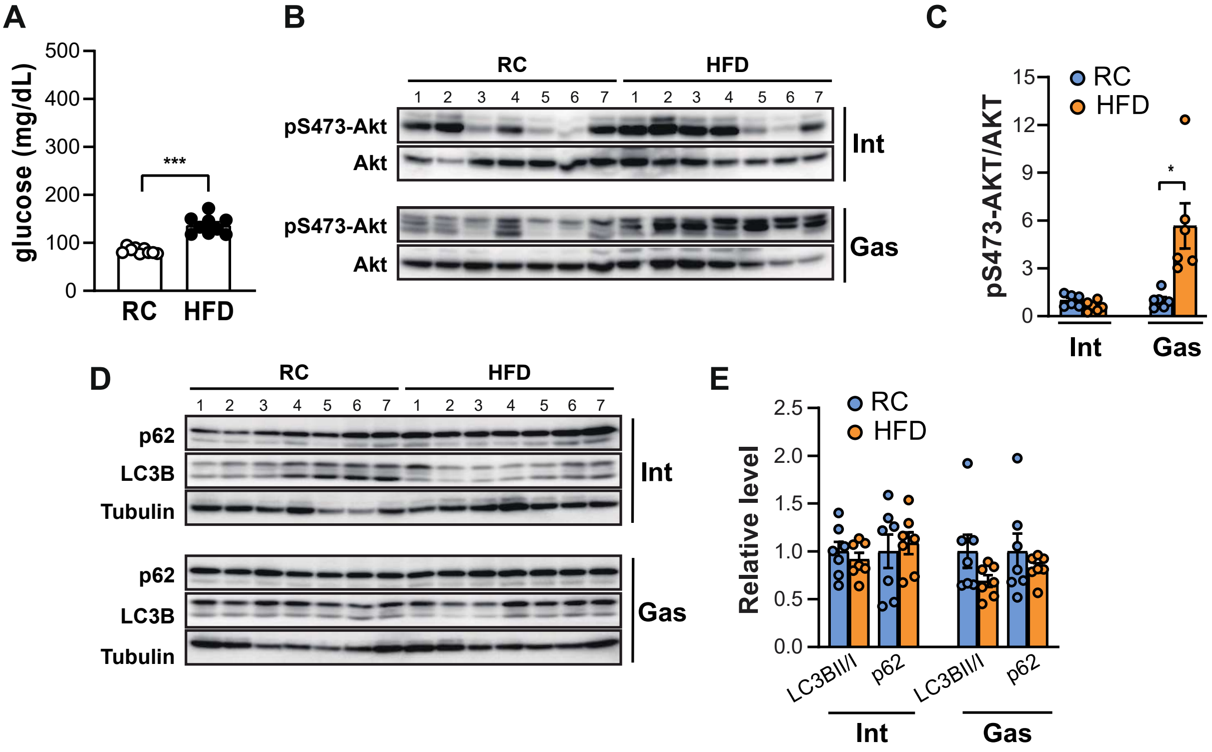

Supplementary Figure S2. Akt phosphorylation and autophagic flux did not change in high fat diet-fed mice. Mice were fed regular chow or a high fat diet for 12 weeks starting at the age of 6 weeks (n = 10–12 per group). (A) Basal blood glucose levels in regular chow or high fat diet-fed mice under normal conditions (n=9). (B-E) The intercostal and gastrocnemius muscles were lysed and subjected to western blot analysis (n=7). (C, E) The relative intensities of the bands were quantified using ImageJ analysis software (n=7). Data are displayed for pSer473-Akt compared to Akt (C), LC3BII compared to LC3BI, and p62 compared to tubulin (E). The data are shown as the mean ± standard error of the mean (E). Abbreviations: regular chow-fed mice (RC); high fat diet-fed mice (HFD); intercostal muscles (Int); gastrocnemius muscle (Gas).

Supplementary Materials and Methods

Diet-induced obesity mouse model

C57BL/6J (Jackson Laboratory, Bar Harbor, ME, USA) were fed a HFD for 12 weeks starting at the age of 6 weeks (n = 10–12 per group). Mice were kept under controlled temperature (22 °C ± 2 °C) with a 12-h light-dark cycle and fed a HFD (60% (kcal) fat diet; D12492 was obtained from Research Diet Incorporation) or regular chow. All experimental protocols for animals were conducted in keeping with Gachon University Animal Care guidelines. All animal procedures were approved by the Center of Animal Care and Use, Lee Gil Ya Cancer and Diabetes Institute, Gachon University and the Institutional Animal Care and Use Committee (IACUC) (Permission number: LCDI-2017-0121).
